# Interpretable ADC-based radiomics models for differentiating hepatocellular carcinoma and intrahepatic cholangiocarcinoma

**DOI:** 10.3389/fonc.2026.1681920

**Published:** 2026-02-03

**Authors:** Yun Zhang, Xiao Yin, Baowen Guo, Hongwu Yang, Zhongjie Huang

**Affiliations:** 1 Department of Radiology, Shenzhen Longhua Maternity and Child Healthcare Hospital, Shenzhen, China; 2 Department of Radiology, Yueyang Central Hospital, Yueyang, Hunan, China; 3 Department of Radiology, The First Affiliated Hospital of Shantou University Medical College, Shantou, Guangdong, China

**Keywords:** apparent diffusion coefficient, hepatocellular carcinoma, imaging radiomics, intrahepatic cholangiocarcinoma, machinelearning

## Abstract

**Objective:**

This study aimed to develop interpretable machine learning (ML) models using apparent diffusion coefficient (ADC) radiomics to differentiate hepatocellular carcinoma (HCC) from intrahepatic cholangiocarcinoma (ICC).

**Methods:**

Radiomic features were extracted from ADC maps of 83 pathologically confirmed HCC and 46 pathologically confirmed ICC patients who underwent MRI examinations. The least absolute shrinkage and selection operator (LASSO) method selected essential features for five ML models: logistic regression (LR), random forest (RF), gaussian naive bayes (GNB), support vector machine (SVM), and k-nearest neighbors (kNN). external validation was performed using 20 HCC and 20 ICC cases from the cancer imaging archive (TCIA) public database. Model performance was assessed using the area under the receiver operating characteristic curve (AUROC), sensitivity, specificity, accuracy, F1 score, calibration plots, and decision curve analysis (DCA). The best-performing model was interpreted using shapley additive explanations (SHAP).

**Results:**

LASSO selected eight features. The models achieved training AUROCs of 0.84-0.95 and internal validation AUROCs of 0.78-0.91. The LR model demonstrated superior performance (training AUROC: 0.95; internal validation AUROC: 0.91; external validation AUROC: 0.85). Moreover, calibration plots and DCA confirmed that this model exhibited the best calibration and clinical utility. SHAP identified wavelet-LLL-firstorder-RootMeanSquared as the most impactful feature.

**Conclusions:**

The ADC-based LR model robustly differentiates HCC from ICC, with validated generalizability using public data, offering a promising non-invasive clinical tool.

## 1 Introduction

Hepatocellular carcinoma (HCC) and intrahepatic cholangiocarcinoma (ICC) are two common but distinct types of liver cancer with different pathological characteristics and clinical presentations (1, 2). HCC originates from hepatocytes, while ICC arises from intrahepatic bile duct epithelial cells, leading to significant differences in pathogenesis, treatment modalities, and prognosis between the two (3, 4). Resection for HCC is based on liver segments, while ICC, being more aggressive in tumor invasion, necessitates resection based on liver lobes (4). Therefore, preoperative differentiation between HCC and ICC is crucial for surgical management. However, clinical differentiation remains challenging,particularly for lesions with ambiguous imaging features or in the setting of multifocal disease. Traditional imaging modalities such as ultrasound, computed tomography (CT), and magnetic resonance imaging (MRI) provide information on tumor morphology and hemodynamics but have limited accuracy and reliability in differentiating HCC from ICC (5).

In recent years, with the rapid development of radiomics techniques, numerous published studies have explored methods for differentiating HCC from ICC using CT and MRI (6–11). For CT, deep learning-based methods such as convolutional neural networks (CNN) have been applied to dynamic contrast-enhanced sequences, extracting high-dimensional features automatically to discriminate liver malignancies, with a reported AUC of 0.92 for distinguishing malignant (including HCC and ICC) from benign/indeterminate lesions (10). For MRI, CNN-based deep learning systems have been developed for multi-phasic sequences (late arterial, portal venous, delayed phases), classifying HCC and ICC with an overall accuracy of 92% for HCC identification (11). Other radiomics methods have leveraged T2WI, contrast-enhanced T1WI, and DWI sequences, quantifying signal intensity variations and tissue microstructure to distinguish the two malignancies, reporting AUCs up to 0.94 (7, 8). However, these methods have limitations. While CT offers good resolution, radiation exposure risks patients and its soft tissue contrast can be poor, limiting microstructural information for radiomics. MRI sequences also have drawbacks: T2WI scans are lengthy, some patients cannot tolerate contrast for T1WI, and DWI signal intensity can be compromised by the T2 shine-through effect (where T2-weighted signal brightness in DWI also appears in apparent diffusion coefficient (ADC) images, potentially causing misdiagnosis (12)), reducing feature extraction accuracy and diagnostic precision. ADC mapping overcomes these shortcomings by quantifying water diffusion restricted by tissue microstructure (e.g., cellular density) (13, 14). ADC reflects tissue water molecule diffusion, directly influenced by tissue microstructure (13). Differences in cellular density, arrangement, and intercellular space between tumor and normal tissue alter ADC values, providing rich microstructural information for tumor differentiation (14). Compared to T2WI and T1WI, ADC typically offers higher spatial resolution for clearer visualization of microstructure (13). Moreover, ADC imaging requires no contrast agent, reducing invasiveness, patient discomfort, and risk, making it more suitable for long-term monitoring or repeat scans than contrast-enhanced T1WI or T2WI. Compared to DWI, ADC is less affected by the T2 shine-through effect (15). ADC more accurately reflects true tissue diffusion, reducing this interference. Consequently, radiomic features extracted from ADC images are likely more accurate and reliable. Critically, existing radiomics-based machine learning (ML) studies suffer from restricted generalizability due to lack of external validation or insufficient interpretability (6–11).

To address these gaps, we developed interpretable machine learning models using ADC radiomics from 83 HCC and 46 ICC patients, with rigorous external validation on 20 HCC and 20 ICC cases from the cancer imaging archive (TCIA) (16)—an open-access database funded by the U.S. national cancer institute. TCIA hosts de-identified cancer imaging data, including liver cancer MRI with ADC sequences and associated clinical annotations. Its publicly available HCC and ICC datasets make it a robust resource for external validation in radiomics-based differentiation studies of these malignancies. LASSO regression selected discriminative features for five ML models ((logistic regression (LR), random forest (RF), gaussian naive bayes (GNB), support vector machine (SVM), and k-Nearest Neighbors algorithm (kNN)), evaluated through comprehensive performance metrics (AUROC, sensitivity, specificity, accuracy, F1 score). Finally, we used the shapely additive explanations (SHAP) package to interpret the best-performing model. SHAP is a game theory-based interpretation framework that attributes each feature’s contribution to a model’s prediction using shapley values. These values indicate how each feature affects the output, making complex model decisions more understandable (17). In medical artificial intelligence (AI), SHAP enhances clinical applicability by providing transparent, individualized explanations for predictions, helping clinicians understand why a model made a certain diagnosis. This fosters trust and facilitates model adoption. SHAP offers both global explanations (highlighting key features across samples) and local explanations (specific to individual predictions), making it ideal for personalized medicine.

## 2 Methods

### 2.1 Study populations

The internal cohort retrospectively included 129 treatment-naïve patients with pathologically confirmed liver malignancies (83 with HCC and 46 with ICC) from our institution and the TCIA between January 2020 and December 2025. Inclusion criteria were: (1) histopathologically confirmed HCC or ICC; (2) dominant lesion diameter≥2 cm; (3) no prior antitumor therapy (including surgery, chemotherapy, radiotherapy, or targeted therapy); (4) available MRI with diffusion-weighted imaging sequences suitable for ADC map reconstruction; (5) minimal motion artifacts on imaging, as independently confirmed by two radiologists with 5 and 8 years of experience in abdominal imaging, respectively. Exclusion criteria were: (1) incomplete clinical or imaging data; (2) concurrent other primary malignancies; (3) severe hepatic or renal dysfunction precluding MRI examination; (4) poor image quality due to technical errors or significant artifacts that interfered with radiomic feature extraction.

Patient characteristics were as follows: in the HCC group, 65 had solitary lesions and 18 had multifocal lesions, with 68 males and 15 females (median age, 60 years; range, 26–84 years); in the ICC group, 32 had solitary lesions and 14 had multifocal lesions, with 24 males and 22 females (median age, 62 years; range, 41–79 years). The internal cohort was randomly split into a training cohort (n=103, 80%) and an internal validation cohort (n=26, 20%) using a stratified sampling method to maintain the original HCC-to-ICC ratio (83:46).

For external validation to ensure the generalizability of the developed models, an independent external cohort of 40 patients (20 HCC and 20 ICC) was retrieved from TCIA. These external cases were selected following the same inclusion and exclusion criteria applied to the internal cohort to minimize selection bias. ADC maps for the external cohort were reconstructed from DICOM-formatted diffusion-weighted imaging data using b-values of 0 and 800 s/mm², consistent with the processing protocol for the internal cohort.

A flowchart summarizing patient inclusion and exclusion is provided in **Supplementary Figure 1**.

### 2.2 MRI acquisition

All MRI examinations for our institution were performed using a single 3.0T Siemens MAGNETOM Trio scanner (Siemens Healthineers, Erlangen, Germany) with DWI sequences acquired in the axial plane. The acquisition parameters were as follows: b-values of 0 and 800 s/mm²; repetition time (TR)/echo time (TE) = 5200/75 ms; acquisition matrix = 112×112; field of view (FOV) = 380×380 mm; slice thickness = 6 mm; interslice gap = 7.2 mm; number of excitations (NEX) = 2.

For the external validation cohort from TCIA, DWI data were retrieved in DICOM format, with acquisition parameters consistent with the internal cohort in terms of key technical specifications (b-values of 0 and 800 s/mm² for ADC map reconstruction). Detailed scanner models and additional sequence parameters for the TCIA data are publicly available in the database metadata, ensuring full transparency of data provenance.

For cross-cohort consistency, z-score normalization was performed to standardize feature distributions, with normalization parameters (mean and standard deviation) derived exclusively from the training set of the internal cohort to prevent information leakage. This normalization scheme was applied consistently to: (i) all samples in the internal cohort (including both training and internal validation subsets); and (ii) the entire TCIA external validation cohort, using the precomputed training set parameters to maintain independence between cohorts.

### 2.3 Feature extraction

ADC maps (DICOM format) were preprocessed using 3D Slicer (v4.11.2, https://www.slicer.org/), with radiomic features extracted via PyRadiomics (v3.0.1, https://pyradiomics.readthedocs.io/). Lesion segmentation was performed double-blinded by two radiologists (Radiologist 1: 10 years of abdominal imaging experience; Radiologist 2: 10 years), both blinded to pathological and clinical data. For well-defined lesions, semi-automatic segmentation (region-growing algorithm) was applied; for those with indistinct margins, manual slice-by-slice delineation was performed. In cases of multifocal disease, the largest lesion was selected as the region of interest (ROI).

Inter-rater reliability was evaluated by comparing the initial segmentations of Radiologists 1 and 2 (excluding consensus cases), quantified using the intraclass correlation coefficient (ICC). An ICC > 0.75 indicated good reproducibility (18), with analyses performed using SPSSAU (v22.0, https://www.spssau.com/).Segmentation discrepancies (Dice similarity coefficient < 0.75) were resolved by a third radiologist (15 years of hepatic imaging experience) via consensus, with the consensus ROI used for subsequent analysis.

A total of 1131 features were extracted, including first-order statistics, shape features, and texture features (e.g., gray-level co-occurrence matrices), as detailed in the PyRadiomics documentation.

### 2.4 Feature selection and dimensionality reduction

Univariate analysis was first performed on radiomic features extracted by Radiologist 1 to screen for those with discriminatory potential between HCC and ICC. The Kolmogorov-Smirnov test was used to assess the normality of feature distributions. For features that followed a normal distribution in both groups, differences between HCC and ICC were compared using independent-samples t-tests; for non-normally distributed features, Mann-Whitney U tests were applied. Features with a *p*-value < 0.05 were considered statistically significant and included in subsequent analyses.

To reduce multicollinearity among the significant features, pairwise Pearson correlation coefficients were computed. When the correlation coefficient between two features exceeded 0.8, indicating high collinearity, only the feature with the most robust discriminatory power (as indicated by the smallest p-value in the univariate analysis) was retained.

Subsequently, LASSO regression was employed for further feature selection, implemented in R software (version 4.3.1; R Foundation for Statistical Computing, Vienna, Austria) via the glmnet package. A 3-fold cross-validation strategy was utilized to determine the optimal regularization parameter (λ) that minimized the mean squared error. The optimal λvalue was found to be 0.07537688, and features with non-zero coefficients at this λ were selected as key predictors for the development of subsequent machine learning models.

### 2.5 Model development and validation

**Figure 1:**
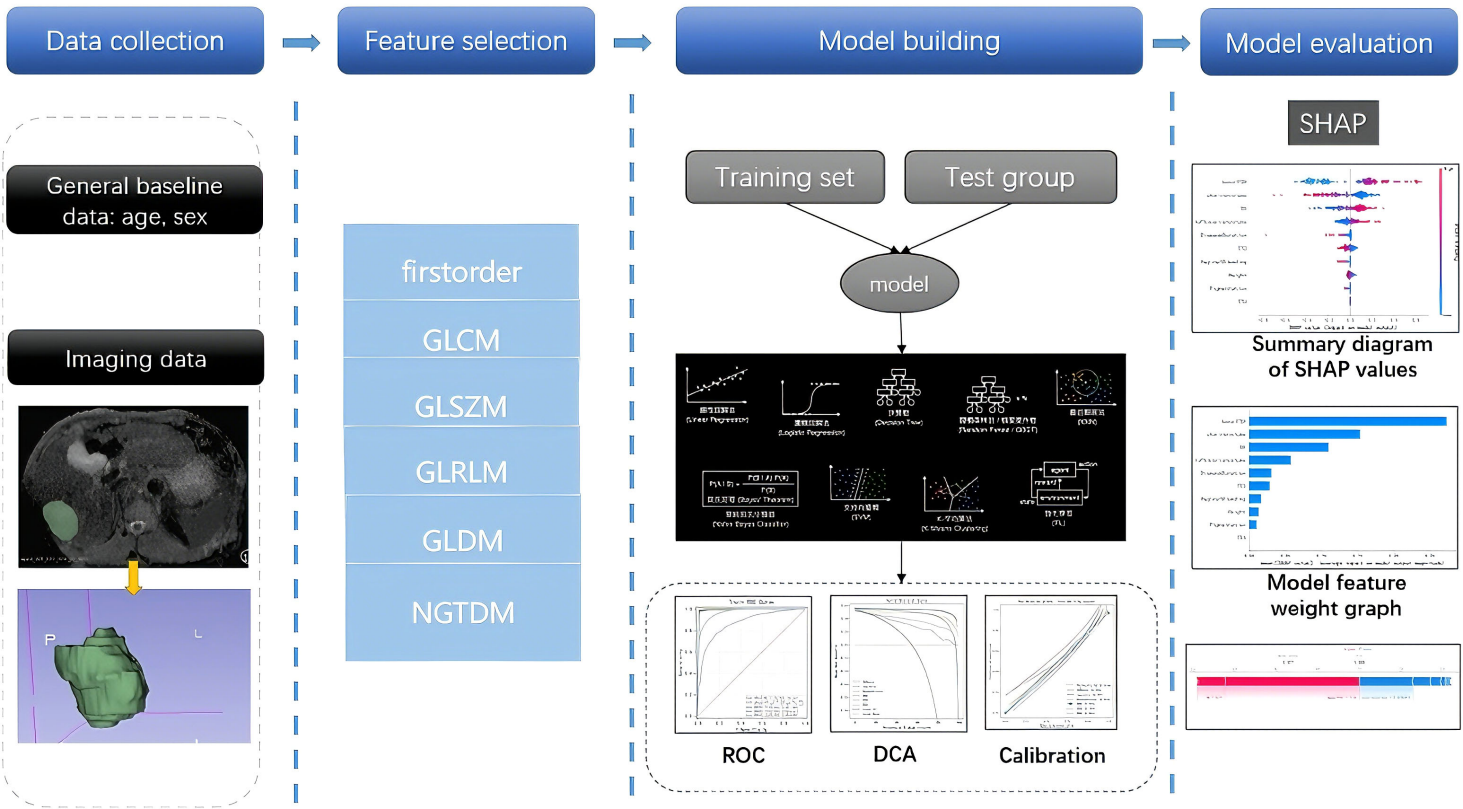
Flow chart for ML models development. GLCM, gray level co-occurrence matrix; GLSZM, gray level size zone matrix; GLRLM, gray level run length matrix; GLDM, gray level dependence matrix; NGTDM, neighbouring gray tone difference matrix; ROC, receiver operating characteristic;DCA, decision curve analysis; SHAP, shapley additive explanations.

To achieve optimal predictive performance, a total of five ML models were developed, encompassing the LR, RF, GNB, SVM, and kNN learning models. Hyperparameters were optimized via 10-fold cross-validation exclusively on the training set. Model performance was evaluated on the locked models (no retraining) using the internal validation set (n = 26) and the external TCIA cohort (n = 40: 20 HCC, 20 ICC). Model discrimination was quantified using the AUROC. Model performance was further evaluated through calibration plots, assessing the extent to which the calibration and model predictions deviated from actual events (19). Clinical utility was assessed using DCA, which calculated net benefits for various threshold probabilities (20). Furthermore, the confusion matrix metrics of AUC, accuracy, sensitivity, specificity and F1 score were assessed for the six models. After the best model was selected, the SHAP package in Python was used to show the relationship between the importance of each parameter. A two-tailed *p* < 0.05 was considered to be statistically significant. The workflow is illustrated in **Figure 1**.

## 3 Results

### 3.1 Patient demographics and tumor characteristics

The clinical and pathological characteristics of the 129 patients (83 HCC, 46 ICC) in the internal cohort are summarized in **Table 1**. The median age was comparable between the HCC and ICC groups (60 vs. 62 years, *p* = 0.321). A significant difference was observed in sex distribution, with a male predominance in the HCC group (81.9%) compared to a more balanced distribution in the ICC group (52.2% male, *p* = 0.002). Although ICC tumors tended to be larger, with a median diameter of 5.1 cm compared to 4.2 cm for HCC, this difference was not statistically significant (*p* = 0.15). Notably, vascular invasion was significantly more frequent in ICC (47.8%) than in HCC (21.7%, *p* = 0.003), a difference driven primarily by a higher rate of microvascular invasion in ICC (41.3% vs. 18.1%, *p* = 0.005).

**Table 1:** Baseline data and tumor characteristics between groups.

Characteristics	HCC (n=83)	ICC (n=46)	P-value
Age, median (range), years	60 (26–84)	62 (41–79)	0.321
Sex, n (%)			0.002
- Male	68 (81.9)	24 (52.2)	
- Female	15 (18.1)	22 (47.8)	
Median tumor size (cm)	4.2 (2.1–12.5)	5.1 (2.3–11.8)	0.15
Tumor size range (cm)	2.1–12.5	2.3–11.8	–
Vascular invasion, n (%)	18 (21.7%)	22 (47.8%)	0.003
Microvascular invasion	15 (18.1%)	19 (41.3%)	0.005
Macrovascular invasion	3 (3.6%)	3 (6.5%)	0.44

#### 3.1.1 Feature extraction consistency

From **Table 2**, it can be observed that the intra-class correlation coefficient range for the 10 cases of extracted image features by radiologist 1 is between 0.871 and 1.000, while the inter-class correlation coefficient range between radiologist 1 and 2 is between 0.921 and 1.000. All correlation coefficients are greater than 0.75, indicating good consistency in feature extraction. Thus, in this study, the influence of subjective factors on lesion segmentation by physicians is minimal, and the repeatability of the study is good.

**Table 2:** Intra-reader and inter-reader reproducibility of radiomic features.

Case	Intra-reader ICC (Radiologist 1)	95%CI	Inter-reader ICC (Radiologist 1 vs 2)	95%CI
1	0.871	0.784∼0.925	0.952	0.916∼0.972
2	0.997	0.995∼0.998	0.921	0.865∼0.954
3	1.000	0.998∼0.999	0.943	0.902∼0.967
4	0.998	0.996∼1.000	0.992	0.985∼0.995
5	0.996	0.993∼0.998	0.973	0.954∼0.985
6	0.998	0.997∼0.999	0.969	0.947∼0.982
7	0.953	0.919∼0.973	0.946	0.907∼0.969
8	0.997	0.996∼0.999	1.000	0.998∼1.000
9	0.998	0.996∼0.999	0.947	0.908∼0.969
10	0.991	0.985∼0.995	0.997	0.994∼0.998

#### 3.1.2 Feature extraction and dimensionality reduction result

**Figure 2:**
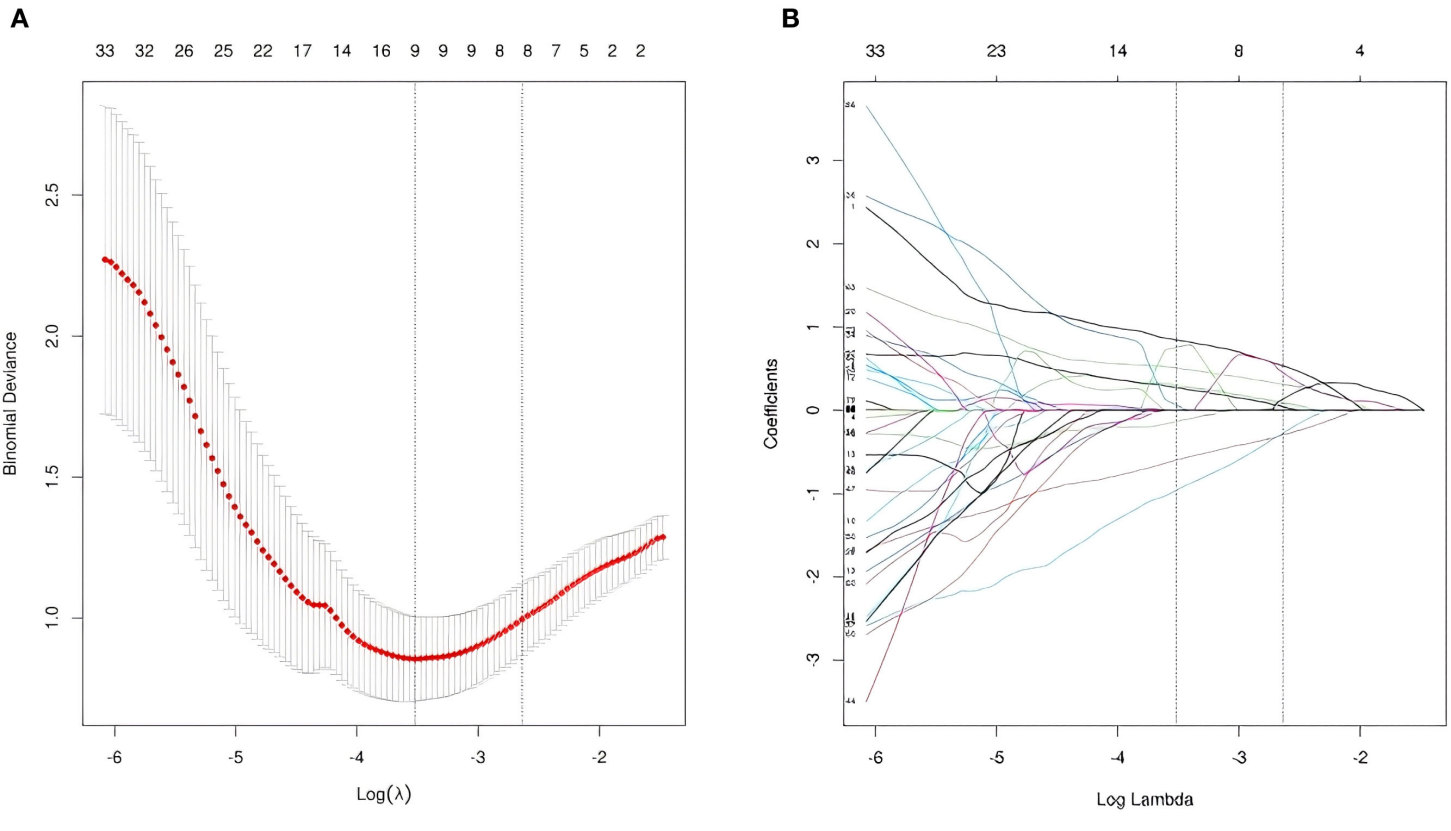
LASSO logistic regression plot. **(a)** Plot of partial likelihood deviance. **(b)** Plot of LASSO coefficient profiles. each colored curve represents the LASSO coefficient profile of a characteristic parameter against the log (λ) sequence.

The 1,131 radiomic features extracted from 169 cases (129 internal + 40 external) using 3D Slicer were categorized into four groups: (1) First-order features (n=252, 22.3%): statistical features based on image intensity histograms, describing pixel value distribution (e.g., mean, variance, skewness, kurtosis) without spatial relationships. (2) Morphological features (n=14, 1.2%): features characterizing tumor geometric shape and structural properties (e.g., volume, surface area, sphericity). (3) Second-order features (n=744, 65.8%): features derived from gray-level co-occurrence matrix (GLCM), gray-level run-length matrix (GLRLM), etc., capturing spatial relationships or texture patterns. (4) High-order features (n=121, 10.7%): multiscale or nonlinear features obtained through filtering or transformations (e.g., wavelet transform, Laplacian transform), reflecting complex imaging patterns. Statistical analysis of 1,131 radiomic features and 2 baseline features (age and sex) revealed 149 radiomic features and sex exhibiting statistically significant differences (*p* < 0.05) between HCC and ICC groups. Subsequent LASSO logistic regression further refined these 150 significantly different features, eliminating redundancy and resulting in 8 meaningful features:sex, original-firstorder-Kurtosis, wavelet-LLL-firstorder-90Percentile, wavelet-LLL-firstorder-RootMeanSquared, wavelet-LLH-firstorder-Skewness, wavelet-LHL-glcm-Correlation, log-sigma-3-0-mm-3D-firstorder-90Percentile, original-shape-Sphericity. The LASSO regression partial likelihood deviation and coefficient profiles against log (λ) are shown in **Figures 2a, b**.

#### 3.1.3 Model development and evaluation results

**Figure 3:**
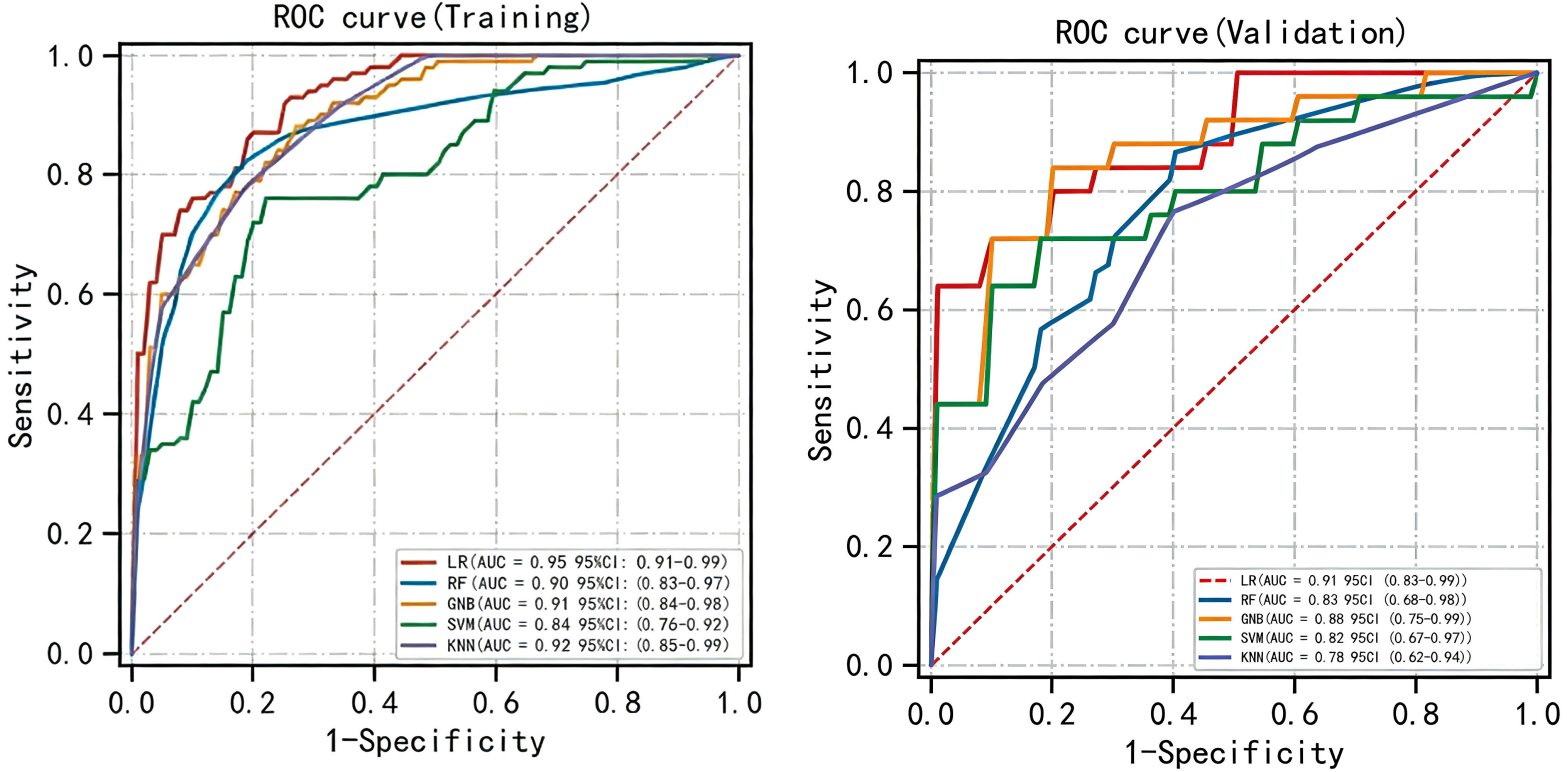
Receiver operating characteristic curves of five ML models for the training cohort (n=103) and internal validation cohort (n=26). CI: confidence interval.

Five machine learning algorithms were used to develop diagnostic models, with the training cohort utilized for model training and hyperparameter optimization. Performance comparisons revealed that all five models achieved robust discriminative ability, with area under the AUROC values ranging from 0.84 to 0.95 in the training cohort and from 0.78 to 0.91 in the validation cohort (**Figure 3**). Among these, the logistic regression (LR) model exhibited the highest AUROC values in both cohorts: 0.95 (95% confidence interval [CI]: 0.91-0.99) in the training set and 0.91 (95% CI: 0.83-0.99) in the validation set (**Figure 3**).

**Figure 4:**
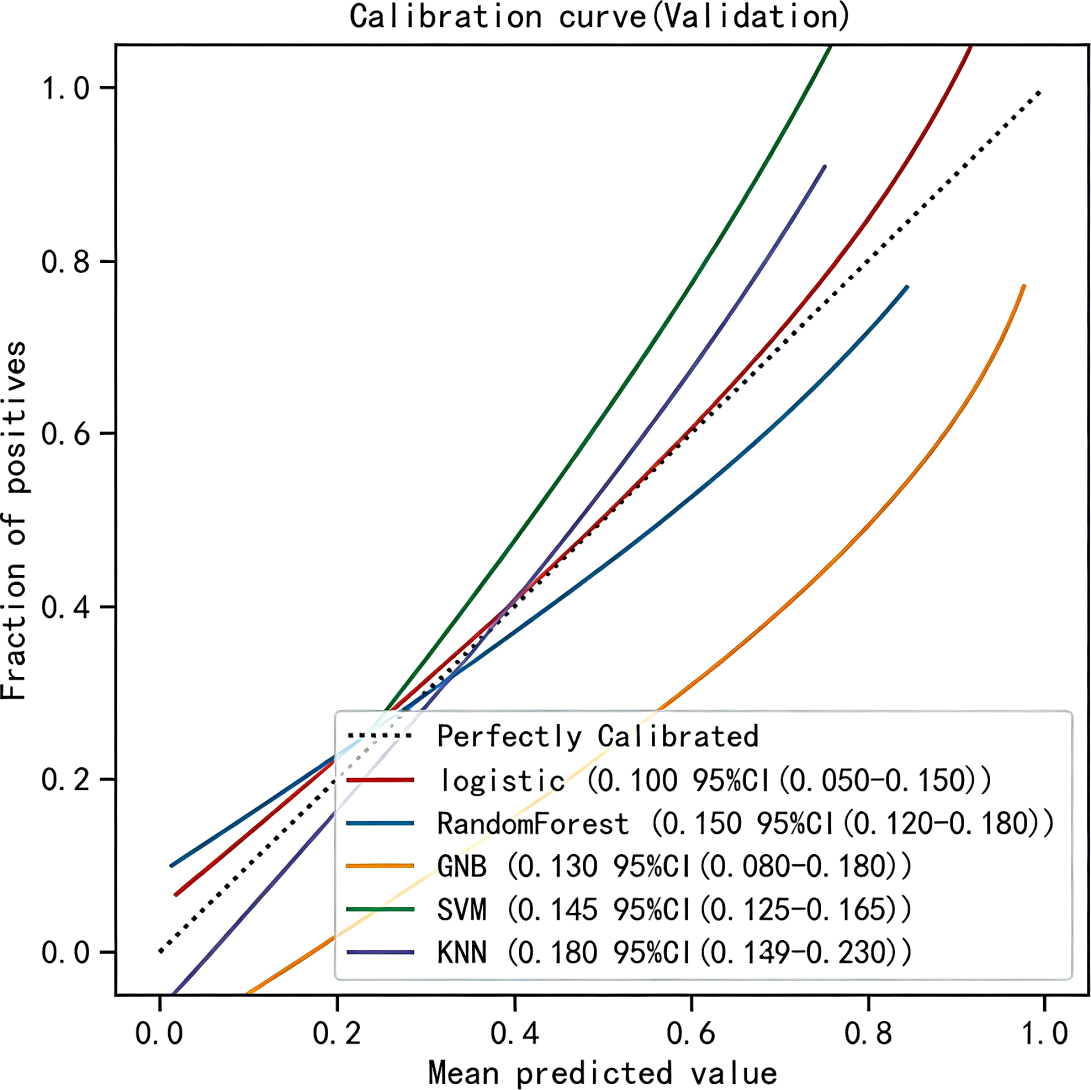
Calibration plots of five ML models. The 45° straight line on each graph represents the perfect match between the actual (y- axis) and predicted (x-axis) probabilities. A closer distance between two curves indicates greater accuracy.

**Figure 5:**
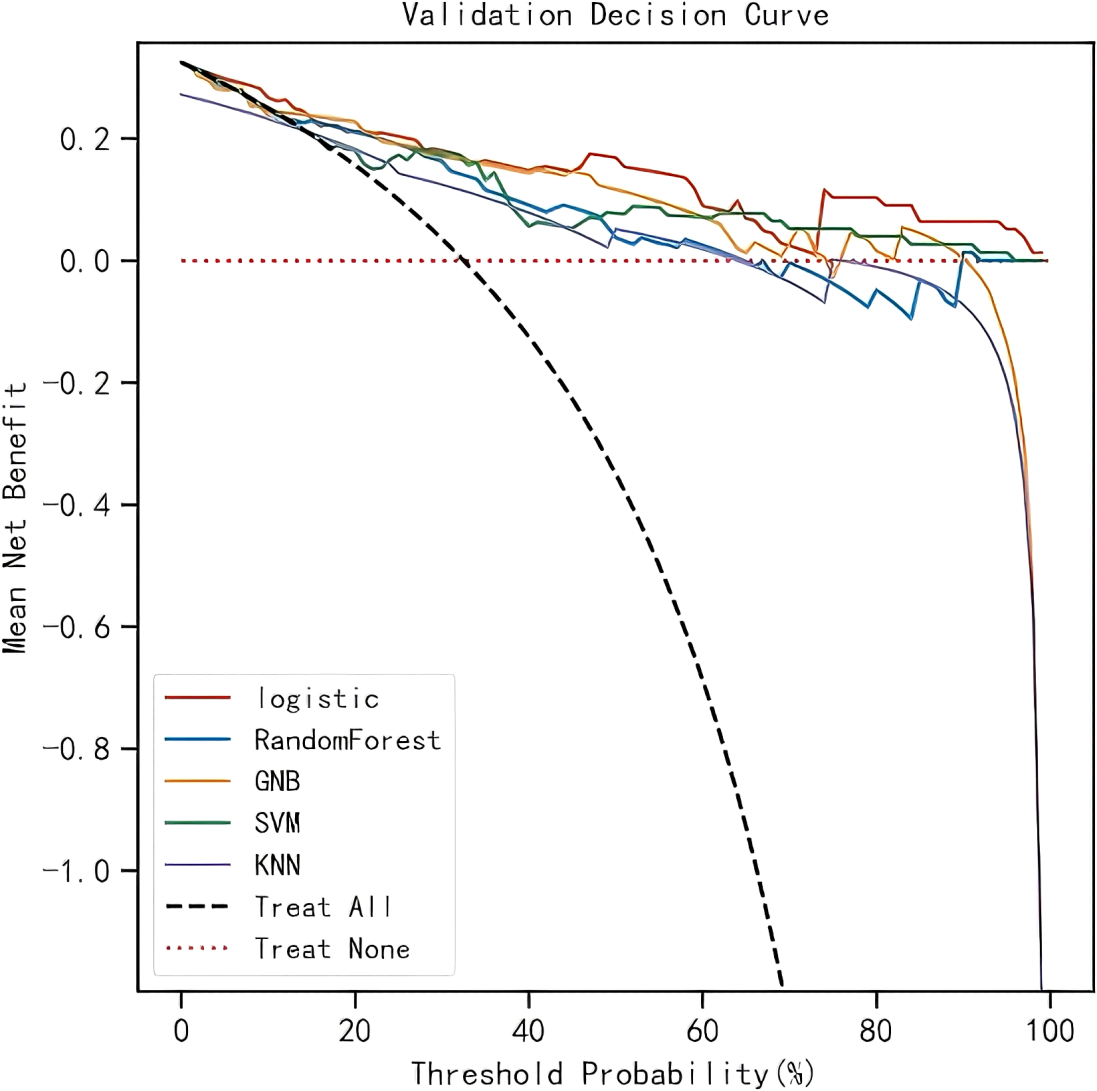
Decision curve analysis for five ML models.

Calibration and clinical utility of the models were further evaluated in the validation cohort. Calibration plots (**Figure 4**) demonstrated that the LR model achieved the lowest brier score (0.100), indicating the smallest discrepancy between predicted probabilities and observed outcomes, thus reflecting optimal calibration. DCA confirmed that all models yielded a net clinical benefit over the “treat-all” or “treat-none” strategies (**Figure 5**), with the LR model exhibiting the highest net benefit across the range of threshold probabilities.

Confusion matrix-derived metrics and optimal diagnostic thresholds for all models in the training and validation cohorts are summarized in **Tables 3**, **4**, respectively. In the training cohort, the LR model outperformed other classifiers with an AUROC of 0.95 and overall accuracy of 0.87. For HCC diagnosis, the model achieved a sensitivity of 0.92 and a specificity of 0.88. For ICC diagnosis, the sensitivity was 0.88 and the specificity was 0.92. The F1 score was 0.85.

**Table 3:** Performance metrics for five models in the training datase.

Model	AUROC (95%CI)	Cutoff (95%CI)	Overall accuracy (95%CI)	HCC sensitivity (95%CI)	HCC specificity (95%CI)	ICC sensitivity (95%CI)	ICC specificity (95%CI)	F1 score (95%CI)
LR	0.95 (0.91-0.99)	0.33 (0.23-0.44)	0.87 (0.84-0.89)	0.92 (0.86-0.98)	0.88 (0.81-0.94)	0.88 (0.81-0.94)	0.92 (0.86-0.98)	0.85 (0.82-0.88)
RF	0.90 (0.83-0.97)	0.40 (0.25-0.54)	0.84 (0.81-0.86)	0.87 (0.80-0.94)	0.83 (0.76-0.89)	0.83 (0.76-0.89)	0.87 (0.80-0.94)	0.82 (0.79-0.84)
GNB	0.91 (0.84-0.98)	0.29 (0.11-0.48)	0.85 (0.82-0.87)	0.86 (0.79-0.93)	0.86 (0.79-0.92)	0.86 (0.79-0.92)	0.86 (0.79-0.93)	0.79 (0.76-0.82)
SVM	0.84 (0.76-0.92)	0.32 (0.29-0.34)	0.80 (0.77-0.82)	0.79 (0.73-0.85)	0.83 (0.77-0.88)	0.83 (0.77-0.88)	0.79 (0.73-0.85)	0.73 (0.70-0.76)
KNN	0.92 (0.85-0.99)	0.44 (0.26-0.62)	0.81 (0.78-0.84)	0.88 (0.81-0.95)	0.78 (0.70-0.85)	0.78 (0.70-0.85)	0.88 (0.81-0.95)	0.83 (0.80-0.85)

LR, logistic regression; RF, random forest; GNB, gaussian naive bayes; SVM, support vector machine; KNN, k-nearest neighbor.

**Table 4:** Performance metrics for five models in the validation dataset.

Model	AUROC (95%CI)	Cutoff (95%CI)	Overall accuracy (95%CI)	HCC sensitivity (95%CI)	HCC specificity (95%CI)	ICC sensitivity (95%CI)	ICC specificity (95%CI)	F1 score (95%CI)
LR	0.91 (0.83-0.99)	0.33 (0.23-0.44)	0.84 (0.72-0.92)	0.90 (0.80-0.98)	0.89 (0.78-0.97)	0.89 (0.78-0.97)	0.90 (0.80-0.98)	0.78 (0.66-0.90)
RF	0.83 (0.68-0.98)	0.40 (0.25-0.54)	0.76 (0.64-0.86)	0.91 (0.81-0.99)	0.71 (0.58-0.83)	0.71 (0.58-0.83)	0.91 (0.81-0.99)	0.74 (0.62-0.86)
GNB	0.88 (0.75-0.99)	0.29 (0.11-0.48)	0.81 (0.69-0.91)	0.83 (0.72-0.94)	0.88 (0.74-1.00)	0.88 (0.74-1.00)	0.83 (0.72-0.94)	0.76 (0.64-0.88)
SVM	0.82 (0.67-0.97)	0.32 (0.29-0.34)	0.79 (0.67-0.89)	0.87 (0.75-0.99)	0.81 (0.66-0.96)	0.81 (0.66-0.96)	0.87 (0.75-0.99)	0.74 (0.63-0.85)
KNN	0.78 (0.62-0.94)	0.44 (0.26-0.62)	0.73 (0.60-0.84)	0.61 (0.45-0.77)	0.86 (0.72-0.98)	0.86 (0.72-0.98)	0.61 (0.45-0.77)	0.57 (0.46-0.68)

LR, logistic regression; RF, random forest; GNB, gaussian naive bayes; SVM, support vector machine; KNN, k-nearest neighbor.

In the validation cohort, the LR model maintained superior performance with an AUROC of 0.91 and overall accuracy of 0.84. For HCC diagnosis, sensitivity was 0.90 and specificity was 0.89; for ICC diagnosis, sensitivity was 0.89 and specificity was 0.90. The F1 score was 0.78. The optimal probability threshold for the LR model, determined by the Youden index, was 0.34.

External validation in the TCIA cohort (n=40: 20 HCC, 20 ICC) confirmed the generalizability of the models (**Table 5**). The LR model demonstrated the highest discriminative ability with an AUROC of 0.85, overall accuracy of 0.84. For HCC diagnosis, sensitivity was 0.83 and specificity was 0.86; for ICC diagnosis, sensitivity was 0.86 and specificity was 0.83. Pairwise comparisons of AUROC values showed that the LR model significantly outperformed other algorithms (all *p* < 0.05).

**Table 5:** External validation performance of five machine learning models on the TCIA cohort.

Model	AUROC (95%CI)	HCC sensitivity (95%CI)	HCC specificity (95%CI)	ICC sensitivity (95%CI)	ICC specificity (95%CI)	Overall accuracy (95%CI)
LR	0.85 (0.72-0.98)	0.83 (0.64-0.95)	0.86 (0.67-0.96)	0.86 (0.67-0.96)	0.83 (0.64-0.95)	0.84 (0.69-0.93)
GNB	0.82 (0.68-0.94)	0.79 (0.60-0.92)	0.81 (0.62-0.94)	0.81 (0.62-0.94)	0.79 (0.60-0.92)	0.80 (0.64-0.91)
RF	0.79 (0.65-0.91)	0.76 (0.57-0.90)	0.78 (0.59-0.92)	0.78 (0.59-0.92)	0.76 (0.57-0.90)	0.77 (0.61-0.88)
SVM	0.76 (0.61-0.89)	0.73 (0.54-0.88)	0.77 (0.58-0.91)	0.77 (0.58-0.91)	0.73 (0.54-0.88)	0.75 (0.59-0.87)
KNN	0.71 (0.55-0.85)	0.67 (0.48-0.83)	0.70 (0.51-0.85)	0.70 (0.51-0.85)	0.67 (0.48-0.83)	0.69 (0.52-0.83)

LR, logistic regression; RF, random forest; GNB, gaussian naive bayes; SVM, support vector machine; KNN, k-nearest neighbor.

### 3.2 Model interpretation

**Figure 6:**
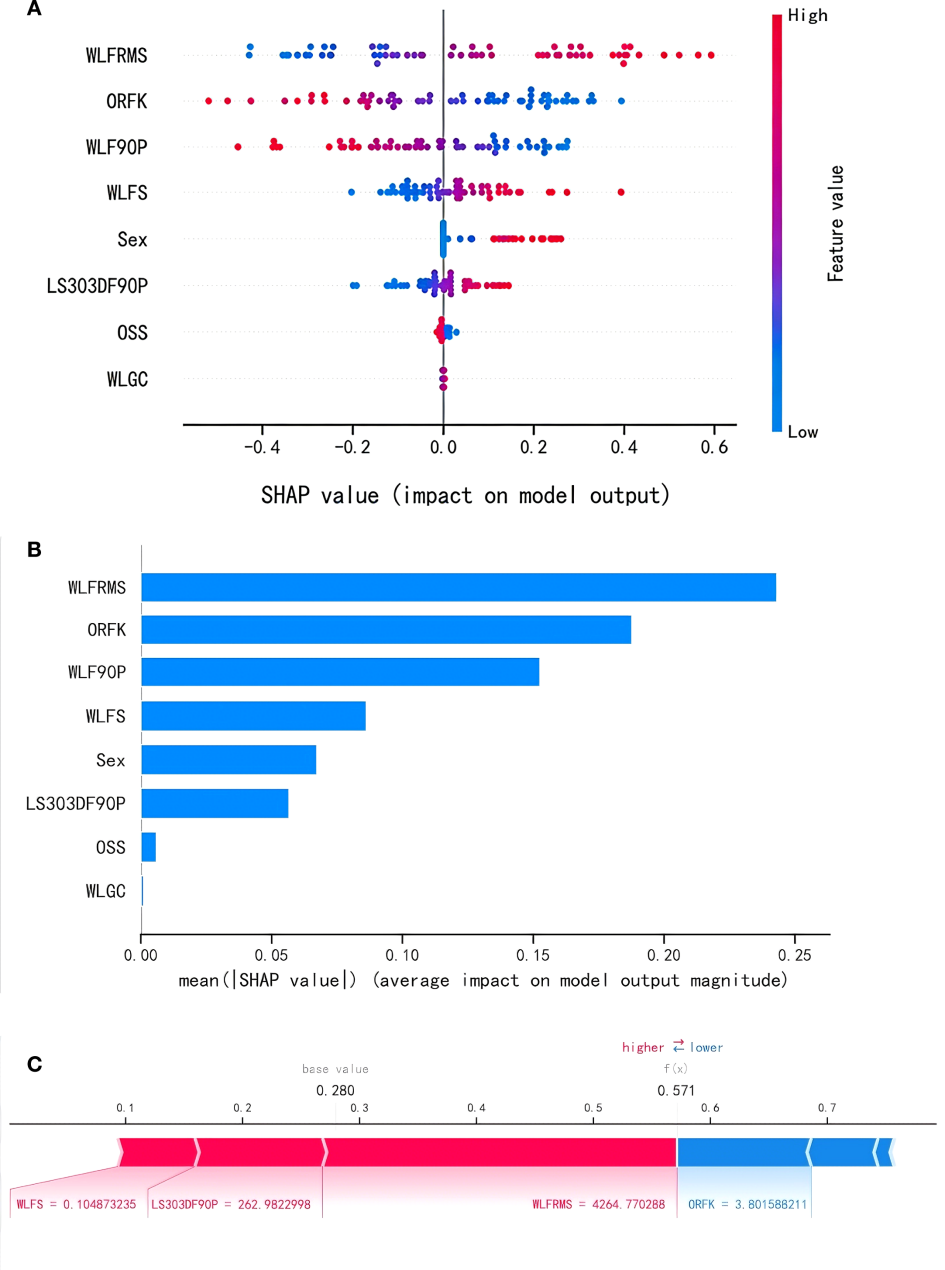
SHAP analysis of the LR model. **(a)** Summary plots of SHAP values. **(b)** Importance plots of characteristic parameter. **(c)** Plots of SHAP explanatory power. **(a)** A visual representation of each feature of the LR model, showing the relationship between the importance of each feature. The color represents the value of the feature, with red representing the larger value and blue representing the smaller value. **(b)** Parameters are arranged along the y axis based on their importance, which is given by the mean of their absolute shapley values. The higher the parameter is positioned in the plot, the more important it is for the model. **(c)** The contributing features are arranged in the horizontal line, sorted by the absolute value of their impact. The output value is the probability of discriminating diagnoses between HCC and ICC. The base value means the expected value of model.ORFK,original-firstorder-Kurtosis; WLF90P,wavelet-LLL-firstorder-90Percentile;WLFRMS,wavelet-LLL-firstorder-RootMeanSquared;WLFS,wavelet-LLH-firstorder-Skewness; WLGC,wavelet-LHL-glcm-Correlation; LS303DF90P, log-sigma-3-0-mm-3D-firstorder-90Percentile; OSS,original-shape-Sphericity.

**Figures 6a, b** represent the SHAP summary plot and feature importance plot for the LR model, respectively. They illustrate the importance ranking of the 8 features and their respective influences on the model’s ability to discriminate between HCC and ICC. **Figures 6a, b** show that wavelet-LLL-firstorder-RootMean.

Squared is the most crucial feature; higher values correspond to higher SHAP values and an increased likelihood of HCC. Similarly, wavelet-LLH-firstorder-Skewness, sex, and log-sigma-3-0-mm-3D-firstorder-90Percentile also exhibit a positive correlation, where higher values correspond to higher SHAP values and an elevated likelihood of HCC. Conversely, original-firstorder-Kurtosis and wavelet-LLL-firstorder-90.

Percentile exhibit a positive correlation with higher SHAP values and an elevated likelihood of ICC. **Figure 6c** provides an example diagnosis for an HCC patient. In this case, four patient features (wavelet-LLH-firstorder-Skewness: 0.10, log-sigma-3-0-mm-3D-firstorder-90Percentile: 262.98, wavelet-LLL-firstorder-Root. MeanSquared: 4264.77, original-firstorder-Kurtosis: 3.80) were input into the model. The model predicted an HCC risk probability of 0.57, which exceeds the LR model’s diagnostic threshold of 0.34, indicating a higher likelihood of HCC for this patient. This suggests that medical staff could prepare for treatment and care in advance.

## 4 Discussion

This study developed and validated interpretable ADC-based radiomics models to differentiate HCC and ICC. Key findings are: (1) ADC radiomics is effective for this task; (2) The LR model demonstrated high internal (AUROC 0.91) and external (TCIA AUROC 0.85) performance; (3) SHAP identified wavelet-LLL-firstorder-Root.

MeanSquared as the most influential feature, enhancing interpretability. Our study addresses the critical clinical challenge of preoperatively distinguishing HCC and ICC, impacting surgical planning. We present a novel, non-invasive tool leveraging quantitative microstructural information from ADC maps, overcoming limitations of operator dependence radiation (CT), and contrast/T2 effects (other MRI). Crucially, robust external validation using TCIA data demonstrates strong generalizability, a key advancement over many radiomics studies. The integration of SHAP interpretation fosters clinical trust by making model decisions transparent.

. Previous studies have explored ML-based differentiation of HCC and ICC using CT (6, 9, 10), and multiphasic MRI (7, 8, 11, 21), reporting AUCs ranging from 0.69 to 0.99 (6–11, 21). Specifically, Yasaka K et al. (10) applied a CNN to dynamic CT, categorizing liver masses into five classes and achieving an AUC of 0.92 in distinguishing malignant from non-malignant or indeterminate lesions. In another CT-based study (9), a modified Inception v3 network was used to classify four common liver tumors (HCC, ICC, CRLM, and benign) from single-phase CT, reaching 96% overall accuracy on a multi-institutional dataset and outperforming experienced radiologists. Furthermore, Hamm CA et al. (11) developed a CNN for multi-phasic MRI that classified six liver lesion types (cyst, hemangioma, FNH, HCC, ICC, and CRC metastasis) with 92% accuracy and an AUC of 0.99 for HCC diagnosis. However, these modalities face limitations: CT entails radiation exposure and offers limited soft-tissue contrast (9, 10); and conventional MRI sequences like T2WI have long scan times, T1WI requires contrast (which some patients cannot tolerate), and DWI suffers from T2 shine-through effects (7, 8, 11, 12), impacting feature reliability. Our study addresses these limitations through two key innovations. First, we leverage ADC maps, which quantify water diffusion restricted by tissue microstructure. ADC provides higher spatial resolution than T2WI/T1WI, requires no contrast or radiation, and minimizes T2 shine-through effects versus DWI (15, 22), enabling more robust feature extraction. Second, we incorporate SHAP for model interpretability, quantifying feature contributions using Shapley values. For example, inputting specific patient features (wavelet-LLH-firstorder-Skewness: 0.10, etc.) yields an HCC risk probability of 0.57 (exceeding the diagnostic threshold of 0.34), indicating higher HCC likelihood (**Figure 6c**). This transparency addresses the “black-box” limitation of prior ML models, enhancing clinical trust and potential for proactive treatment planning pending validation.

The LR model harnessed eight discriminative predictors derived from ADC maps, each capturing distinct yet complementary aspects of the underlying pathobiology that enables HCC and ICC differentiation. The most crucial feature, wavelet-LLL-firstorder-RootMeanSquared, measures overall tumor density: ICC’s dense cellular/fibrous tissue lowers water diffusion (reducing this value), while HCC’s looser structure increases it. This density difference is complemented by original-firstorder-Kurtosis, where HCC’s frequent necrosis/bleeding creates irregular signal peaks (higher values) versus ICC’s uniform fibrosis. Signal distribution asymmetry (wavelet-LLH-firstorder-Skewness) may indicate uneven barriers like fibrosis patterns. Concurrently, wavelet-LLL-firstorder-90Percentile and log-sigma-3-0-mm-3D-firstorder-90Percentile reveal where water moves freely, highlighting differences in edematous/microvascular regions. Texture analysis via wavelet-LHL-glcm-Correlation shows ICC’s organized fibrous bands create regular patterns (higher values), contrasting with HCC’s chaotic structures from necrosis/blood pools (lower values). Morphologically, original-shape-Sphericity confirms ICC’s irregular, infiltrative shapes (lower values) versus HCC’s rounder nodules (higher values). Finally, sex incorporates epidemiology: male predominance in HCC (viral/alcohol risks) and female tendency in ICC (biliary diseases). Together, these features synergistically detect ICC through denser tissue, uniform structure, irregular shape, and female association, while identifying HCC via looser tissue, internal heterogeneity, chaotic textures, spherical form, and male prevalence - with tumor density remaining the primary discriminator.

This interpretable ADC-based LR model offers significant clinical potential by providing a rapid (e.g., <5 min), non-invasive, and objective tool for preoperative HCC vs. ICC differentiation directly from routine MRI sequences. Its high performance (AUROC 0.85 externally) and generalizability support its use in triaging indeterminate lesions (2+ cm), particularly where biopsy is risky or advanced imaging is unavailable. Crucially, SHAP explanations enhance trust and facilitate communication in multidisciplinary tumor boards. Notably, the LR model exhibited balanced and robust performance for both HCC and ICC individual diagnosis. For HCC, the sensitivity reached 0.92 (training), 0.90 (internal validation), and 0.83 (external validation), indicating high efficiency in identifying true HCC cases and minimizing false negatives—critical for avoiding missed diagnosis of HCC which requires segmentectomy. For ICC, the sensitivity was 0.88 (training), 0.89 (internal validation), and 0.86 (external validation), with specificity of 0.92 (training), 0.90 (internal validation), and 0.83 (external validation), ensuring accurate recognition of ICC (which necessitates lobectomy) and reducing false positives. This balanced diagnostic performance for both malignancies addresses the clinical need for precise preoperative differentiation, as misclassification of either tumor type could lead to inappropriate surgical strategies. By leveraging existing ADC maps without requiring contrast or radiation, the model reduces costs, eliminates contrast-related risks, and minimizes unnecessary surgeries, enabling optimized surgical planning (e.g., segmentectomy for HCC vs. lobectomy for ICC) based on non-invasive prediction. Initial integration into clinical workflows is feasible using standard DICOM data, though local validation of the probability threshold is recommended.

Our study has several limitations. Firstly, lesions < 2 cm were excluded due to technical difficulties in radiomic feature extraction—these small lesions are precisely the most challenging scenarios in clinical diagnosis, which may limit the model’s applicability to early-stage liver malignancies. Secondly, despite z-score normalization mitigating technical heterogeneity, vendor-specific ADC computation algorithms across MRI scanners (e.g., internal Siemens vs. multi-institutional TCIA systems) may affect feature reproducibility, highlighting the need for further validation across more uniform imaging platforms. Lastly, this study adopts asingle-center retrospective design, which may introduce inherent selection bias and limit the generalizability of the findings to broader populations.

## Conclusions

We developed and externally validated interpretable machine learning models based on ADC map radiomics for the differential diagnosis of HCC and ICC. The Logistic Regression model demonstrated high and generalizable performance (Internal AUROC 0.91, External TCIA AUROC 0.85). SHAP analysis provided crucial interpretability, identifying wavelet-LLL-firstorder-RootMeanSquared as the most important feature. This study establishes ADC radiomics as a robust, radiation-free, and contrast-free approach for non-invasively distinguishing these two common primary liver cancers. The validated model serves as a reliable diagnostic aid, providing objective information critical for clinical management planning.

## Data Availability

The raw data supporting the conclusions of this article will be made available by the authors, without undue reservation.
