# Supplementary figures and images for: Interpretable ADC-based radiomics models for differentiating hepatocellular carcinoma and intrahepatic cholangiocarcinoma
Source: Front Oncol. 2026 Feb 3;16:1681920. doi: 10.3389/fonc.2026.1681920 (PMC12909249; doi:10.3389/fonc.2026.1681920)

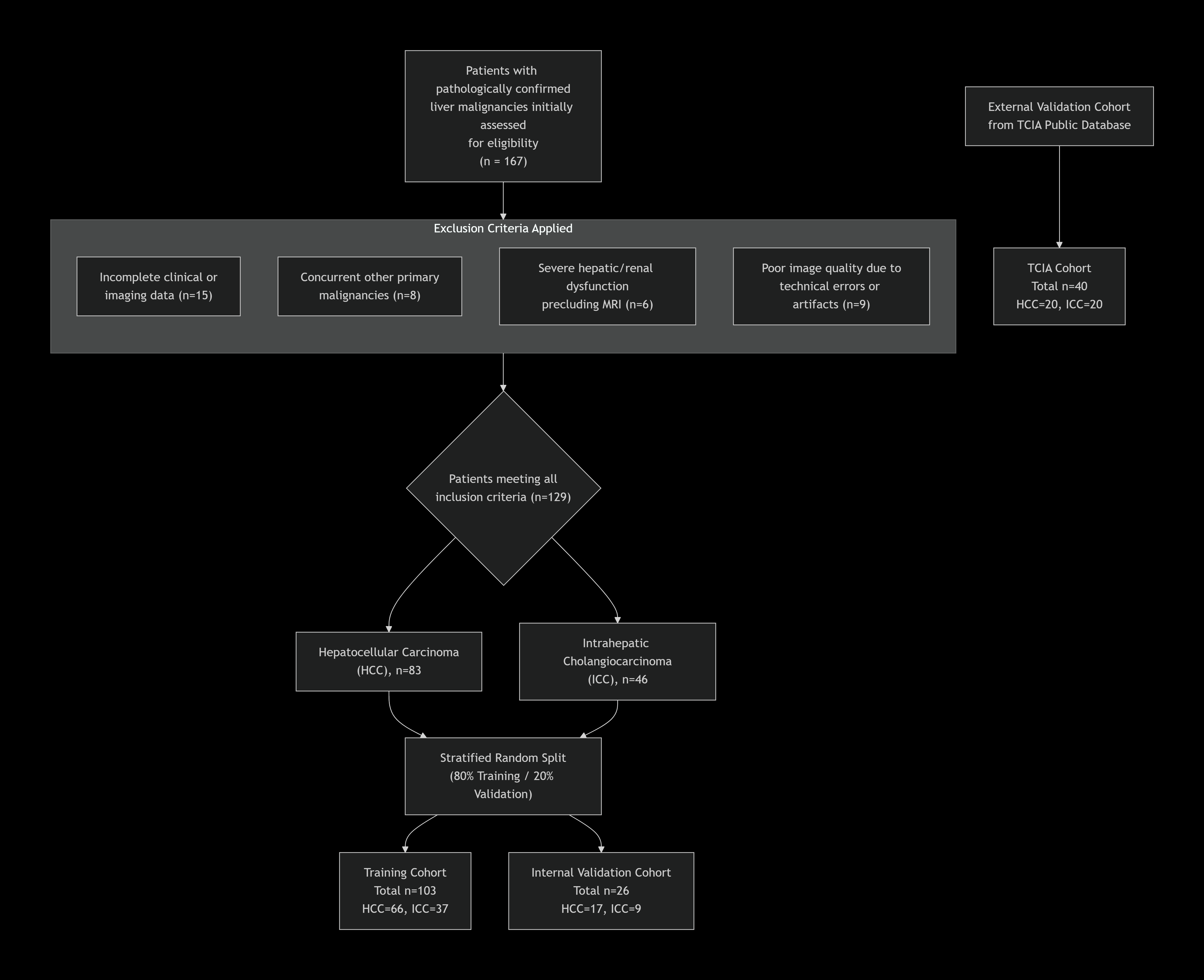

Supplement: Supplementary file 1 [file DataSheet1.zip › Supplementary Figure 1.tif]
